# Impact of *PaGLK* transgenic poplar on microbial community and soil enzyme activity in rhizosphere soil

**DOI:** 10.3389/fbioe.2022.965209

**Published:** 2022-07-22

**Authors:** Yu Zheng, Guan Bin Lv, Kun Chen, Qibin Yu, Ben Niu, Jing Jiang, Guifeng Liu

**Affiliations:** ^1^ State Key Laboratory of Tree Genetics and Breeding (Northeast Forestry University), Haerbin, HL, China; ^2^ Citrus Research and Education Center, University of Florida, Lake Alfred, FL, United States

**Keywords:** transgenic, rhizosphere, soil enzyme activity, community composition, metabolite

## Abstract

Rhizosphere microorganisms are essential parts in maintaining soil ecological functions. Reforestation using genetically modified trees might have great potential to enhance tree production in biotic and abiotic stress, however, their long-term impact on rhizosphere microorganisms is scant. In this study, we studied soil enzyme activities and composition of rhizosphere microorganisms in 2-year-old transgenic *PaGLK* overexpression (OE), repressed expression (RE) and wild-type (WT) poplar (*P. alba × P.berlinensis*). The root exudates of *PaGLK* transgenic poplar (*P.alba × P. berlinensis*) were analyzed by liquid chromatography-mass spectrometry (LC-MS). The results showed that there were significant difference for soil sucrase, urease, catalase, neutral protease and cellulase between the transgenic and WT lines at different growth periods. Alpha diversity analysis showed that bacterial community abundance and diversity for RE lines were significantly lower than WT (*p* < 0.05), while RE lines for fungi were significantly higher than WT lines. At the genus level, *Burkholderia* was the dominant group of rhizosphere bacterial community, and the relative abundance for RE was significantly higher than WT. *Tomentella* was the dominant group for fungi community. *Serendipita* for RE was significantly higher than WT and OE. Main metabolite contents of (S)-ACPA, geniposidic acid, agnuside, hydroquinone and pyranocoumarins were significantly different among transgenic lines. These results suggest that transgenic activities have effects on root exudates, rhizosphere soil enzyme activities and soil microbial community composition, but long term effects need to be further investigated.

## Introduction

Soil microorganisms maintain soil ecological functions and play an important role in participating plant growth, soil nutrients and formation of humus. In rhizosphere, soil microorganisms and plant roots are closely interacted in competitive and cooperative way to form a complex equilibrium relationship (Philippot et al., 2013). Plants produce organic matters by photosynthesis and release them into the soil in the form of root exudates or mucus to provide nutrients for soil microorganisms; whereas rhizosphere soil microorganisms decompose the organic matters released by plants and return them to the soil in the form of inorganic matter, which promotes the nutrient cycle of the ecosystem (Lynch&Whipps., 1990).

In recent years, the reforestation of genetically modified trees has become a controversial topic of interest, due to concerning foreign gene expression products altering enzymic activity of rhizosphere soil and microbial community composition, Previous studies have been reported in transgenic maize, cotton and other crops, and the effects of foreign gene products on rhizosphere soil microorganisms were inconclusive (Yasin et al., 2016; Ahamd et al., 2017; van Wyk et al., 2017). Some transgenic plants inhibit the growth of some microorganisms in the soil through root exudates, and promote the survival and reproduction of other microorganisms, resulting in favorable soil for plant growth (Phour et al., 2020). *Bt* transgenic cotton (*G. hirsutum*), CrylAc + CpTI transgenic cotton and Cry1Ie transgenic maize (*Zea mays* L.) showed that there were no significate differences for diversity and composition of rhizosphere soil microbial community with non-transgenic plant (Zhang et al., 2015; Jingang et al., 2018; Li et al., 2018). In forestry, transgenic genetic poplar, such as insect resistant transgenic poplar, has been approved and become possibility of deployment (Jing et al., 2004; Guifeng et al., 2006; Guifeng et al., 2006; Zhou et al., 2018). More transgenic poplars are still in the stage of trial and environmental risk assessment (Lu et al., 2014; Zhu et al., 2016; Hu et al., 2017). Five years of field monitoring of the *Bt* transgenic 741 poplar showed that the introduction of the *Bt* gene has neither affected the stability of arthropod communities nor the rhizosphere soil physicochemical properties and microbial community structure (Zuo et al., 2018). Other studies on transgenic poplar such as transgenic *Bt* Poplar and transgenic *populus × euramericana* ‘Guariento’ showed that the introduction of exogenous sources had limited effect on the composition of rhizosphere soil bacterial or fungal communities (Bonadei et al., 2010; Fan et al., 2020; Huang et al., 2022).

In our previous study, we used the male strain of a male triploid hybrid poplar, *P. alba × P. berlinensis* to produce *PaGLK* overexpression (OE) and repressed expression (RE) lines by *Agrobacterium* mediated leaf disk method (Li et al., 2021). Golden 2-like (GLK) is mainly involved in plant chloroplast development, affecting leaf color or other functions (Yeh et al., 2022). Leaf chlorophyll content in OE was significantly higher, while leaf chlorophyll content in RE was significantly lower than the WT. Leaf color of RE lines was bright yellow green. Therefore, we expect that RE could become a new cultivar for urban ornamental tree. As GMO regulatory requirements, we need to carry out biosafety evaluation for the transgenic *PaGLK* poplar. In this study, we characterized the soil enzyme activity and microbial community in the rhizosphere soil, in order to define the impact of transgenic *PaGLK* poplar hybrid (*P. alba × P. berlinensis*) on rhizosphere microorganisms.

## Materials and methods

### Plant materials

The Wild type poplar (WT) material used is *P.alba × P.berolinensis,* grown on MS solid medium with MS + 0.5 mg/L 6-BA + 0.02 mg/L NAA. The transgenic poplar plants overexpressing *PaGLK* (OE1, OE2, and OE3, hygromycin as a selection agent) grown on MS solid medium with MS+0.5 mg/L 6-BA+0.02 mg/L NAA+200 mg/L Cef+10 mg/L Hyg. The transgenic poplar plants repressed expression *PaGLK* (RE1, RE2 and RE3, Glufosinate ammonium as a selection agent) grown on MS solid medium with MS + 0.5 mg/L 6-BA + 0.02 mg/L NAA + 200 mg/L Cef + 1 mg/L Glufosinate ammonium. The above tissue culture subculture plants were preserved in our laboratory. The rootless seedlings were cultured in the rooting medium (1/4 MS + 0.5 mg /L IBA) for about 30 days. The seedlings were transplanted to the seedling tray in mid-April 2019. The plant substrate was soddy soil: black soil: vermiculite (v/v) = 4:3:3, which was placed in the plastic shed for conventional water and fertilizer management. The highly consistent plants were selected in mid-May and transplanted to the 21 cm × 21 cm flower pot. The substrate was soddy soil: river sand: black soil (v/v) = 2:1:1, and the plastic tray was placed under the pot. A total of 40 plants grew at the Birch Breeding Base located at 126°64′E and 45°72′ N of Northeast Forestry University in Harbin, Heilongjiang Province. It belongs to the continental monsoon climate in the middle temperate zone. The annual average precipitation is 569.1 mm. The precipitation is mainly concentrated from June to September, and the frost free period is 168 days. There are four distinct seasons. The annual average temperature is 5.6°C, the highest monthly average temperature is 23.6°C, and the lowest monthly average temperature is −15.8°C.

### Soil sampling

Soil samples were collected from rhizosphere of transgenic OE lines (OE1, OE2, OE3), RE lines (RE1, RE2, RE3) and WT lines. Sampling was done on the day 15th in each month of July, August and September 2020. Each line had three biological replications with a total of 21 plants. Soil samples with three samples per plant were collected under 5cm from soil surface. Soil samples were air-dried at laboratory for further analysis of soil enzyme activity. Plant samples were collected on 3 September 2020, 21. After removing the floating soil, the manual soil sampler was used to collect the roots with soil down to the middle layer of the flowerpot. Three places were sampled in each pot. The loose soil was gently shaken off, and evenly mixed for testing. The roots with soil attached were put into zipper bags before measuring the composition of rhizosphere microbial community.

### Determination of soil enzyme activity

Soil enzyme activity was determined by spectrophotometric method. The air-dried soil samples were passed through a 50-mesh sieve, and the assay tubes and standard tubes were set up using soil urease (S-UE), soil cellulase (S-CL), soil catalase (S-CAT), soil sucrase (S-SC) and soil neutral protease (S-NPT) kits (Suzhou Comin Biotechnology Co., Ltd.), and finally, the extracted supernatant was measured at the wavelengths of 578, 620, 240, 510 and 680 nm respectively, and repeated for 3 times. The values were counted in Excel and calculated as graphs, and SPSS software was used for ANOVA and multiple comparisons.

### Determination of rhizosphere microbial community composition

Root sample was placed in 0.85% NaCl sterile solution triangular flask, shaking at 200 rpm/min for 15 min, then the root was discarded. Soil suspension in 2 ml was collected in centrifuge tube and centrifuged at 10000 rpm / min at 4°C for 10 min. After discarded supernatant, soil precipitation was collected with 6 tubes per sample. Total DNA of soil microorganisms was extracted from soil sample by DNeasy PowerSoil Kit (QIAGEN Group). High-throughput sequencing was done by Shanghai Personal Biotechnology Co.,Ltd. for bacterial 16S and fungal ITs database construction. DADA2 method was used for primer removal, quality filtering, denoising, splicing and chimerism removal. Each de duplication sequence generated after quality control was called ASVS (amplitude sequence variants). QIIME2 (2019.4) was used for alpha diversity and species composition analysis.

### Determination of root exudates

The shoots collected from tissue culture transgenic OE, RE and WT plants were cultured in the rooting medium. After 25 days, the shoots with the same growth status (2 plants per tube, 15 tubes per line) were collected in 50 ml centrifuge tubes. Then, 15 ml sterile deionized water was added and cultured at 25 ± 2°C for 30 days under light/dark cycle of 16 h/8 h. The culture solution was collected in every 10 days, and another 15 ml of sterile deionized water was added. Then, the collected root culture solution was stored at −80°C. After three collections, samples were sent to Shanghai Hoogen Biotechnology Co., Ltd. for GC-MS analysis. As for chromatographic condition, ACQUITY UPLC BEH C18 column (100 mm × 2.1 mm, 1.7 μm, Waters, United States) was used. For mass spectrometry, electrospray ion source (ESI) and data were collected in positive and negative ion modes. The samples were separated by UHPLC and analyzed by Q-Exactive quadrupole-electrostatic field orbital trap high resolution mass spectrometer (Thermo Fisher Scientific, United States).

## Results

### Enzyme activity in rhizosphere soil of transgenic poplar

**FIGURE 1:**
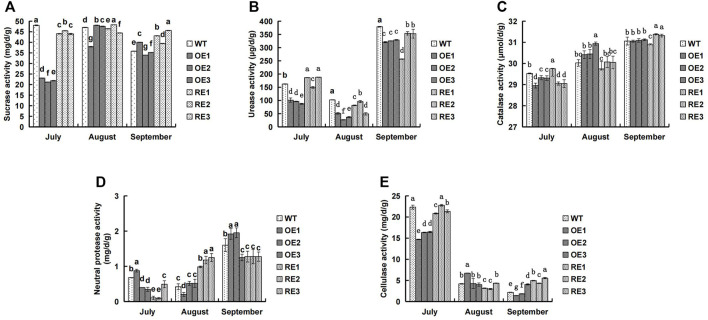
Comparison of rhizosphere soil enzyme activities between transgenic lines and WT. **(A)** Sucrase activity **(B)** Urease activity **(C)** Catalase activity **(D)** Neutral protease activity **(E)** Cellulase activity.

The five enzyme activities in rhizosphere soil of transgenic *PaGLK* poplar OE, RE lines, and WT were presented in Figure 1. In mid-July, the five soil enzyme activities in rhizosphere soil of OE and RE lines were lower than those of the WT, however, there were few exceptions: urease activities for RE1 and RE3, catalase activities for RE1, cellulase activities for RE2, and neutral protease activities for OE1. In particular, the rhizosphere soil enzyme activities of OE lines were the lowest, except neutral protease activities of OE1. Sucrase and urease for OE were 54.11% and 41.46% lower than WT, respectively. In general, the average activity of OE was lower than WT. The catalase and cellulase were 1.12% and 29.09% lower than WT, respectively. As plant grew, cellulase decreased. However, other four soil enzyme activities showed an increasing trend.In mid-August, the urease activity (Figure 1B) of the transgenic lines was significantly lower than WT, the catalase activity (Figure 1C) of the OE showed a higher trend than WT, and the neutral protease activity (Figure 1D) of the RE was significantly higher than WT.

### Characteristics of bacterial and fungal communities in rhizosphere of transgenic poplar

**FIGURE 2:**
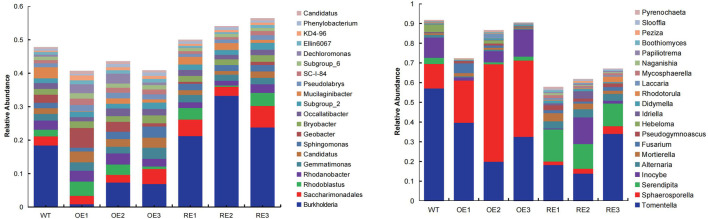
Relative abundance of rhizosphere soil microbial communities in populus in the first 20 orders. **(A)** Bacteria **(B)** Fungi.

The diversity of microbial community of rhizosphere soil for transgenic poplar are shown in Figure 2. There are 971 genera for bacterial communities in the rhizosphere (Figure 2A). *Burkholderia* was a dominant group for all transgenic lines, except OE1. The relative abundance of this genus in RE lines was significantly higher than WT, while the relative abundance of OE lines was significantly lower than WT.

There were 466 genera in the rhizosphere soil fungal community of the tested lines at the genus level (Figure 2B). *Tomentella* was a dominant group of the rhizosphere fungal community for all transgenic lines. *Tomentella* for WT was 57.13% which was the highest, while *Tomentella* for OE and RE decreased to varying degrees (Figure 2B). In addition, it was also found that *Sphaerosporella* for OE increased significantly, and the average value was 24.20% higher than WT and 33.83% higher than RE. *Serendipita* for RE lines was significantly higher than the WT and OE.

### Changes of bacterial and fungal community abundance and diversity in rhizosphere soil

Table 1 showed that the sequencing coverage of bacteria and fungi for all samples were above 96%. Results also showed that the diversity changes of bacterial and fungal communities in rhizosphere soil. The observed species, Chao1 community abundance parameters, Shannon and Simpson diversity indexes showed that there was no significant difference in bacterial community abundance and diversity between OE and WT (except OE2). However, bacteria community abundance and diversity for RE lines were lower than WT (*p* < 0.05). In terms of fungal community composition, the abundance and diversity for OE and RE lines were higher than those of WT lines, except OE3.

**TABLE 1:** Alpha diversity of bacteria and fungi in rhizosphere soil of transgenic *PaGLK* poplar.

Class	Sample	Observed_species	Chao1	Shannon	Simpson	Coverage
Bacteria	WT	4793a	6015a	9.967ab	0.9912a	0.9694b
	OE1	4863a	5826ab	10.31ab	0.9962a	0.9722ab
	OE2	4266bc	5078bc	9.849ab	0.9903a	0.9769a
	OE3	4497ab	5336abc	10.30a	0.9974a	0.9756a
	RE1	3911c	4975c	9.279bc	0.9856a	0.9758a
	RE2	3768c	4741c	8.717c	0.9632b	0.9773a
	RE3	4097bc	5125bc	9.443bc	0.9870a	0.9742ab
Fungi	WT	465cd	465.60cd	3.791cd	0.8235ab	0.9999a
	OE1	655ab	656.03ab	4.609bc	0.8402ab	0.9999a
	OE2	536bcd	537.73bcd	3.828cd	0.7221bc	0.9999a
	OE3	369d	370.77d	2.623d	0.6238c	0.9999a
	RE1	721a	722.35a	6.045a	0.9417a	0.9999a
	RE2	634abc	635.49abc	5.654ab	0.9397a	0.9999a
	RE3	592abc	593.06abc	5.906ab	0.9512a	0.9999a

Note: Means with the same letters were not significantly different at *p* < 0.05.

**FIGURE 3:**
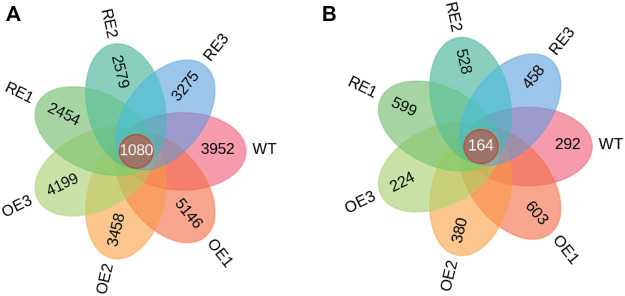
ASV Wayne diagram of bacteria and fungi in rhizosphere soil of *P. alba × P. berlinensis.* **(A)** Bacteria **(B)** Fungi.

Wayne diagrams were based on the characteristic sequences (ASV) of bacteria and fungi in rhizosphere soil among the tested lines (Figure 3). As for rhizosphere soil bacteria, ASV was only 2.94% of the total 1080 in the tested lines. This indicates that there were significant differences of ASV among tested lines (Figure 3A). The specific ASV of WT lines was 3952. The specific ASVs of the three OE lines were 5146, 3458 and 4199, respectively. The specific ASVs for RE lines were 2454, 2579 and 3275, respectively. According to ASVs shared with the WT, the highest similarity with WT was 306 for OE1, and the lowest similarity was 215 for RE3.

As for rhizosphere soil fungi, there were 164 ASVs in all tested lines which was accounted for 3.54% of the total. This indicates that there were differences in ASVs among the tested lines (Figure 3B). The specific ASV of WT lines was 292; the specific ASVs of the three OE lines were 603, 380 and 224, respectively; the three specific ASVs of RE lines were 599, 528 and 458, respectively. According to the number of ASVs shared with WT, the highest similarity with WT was 35 for OE1, and the lowest similarity was 12 for OE3.

**FIGURE 4:**
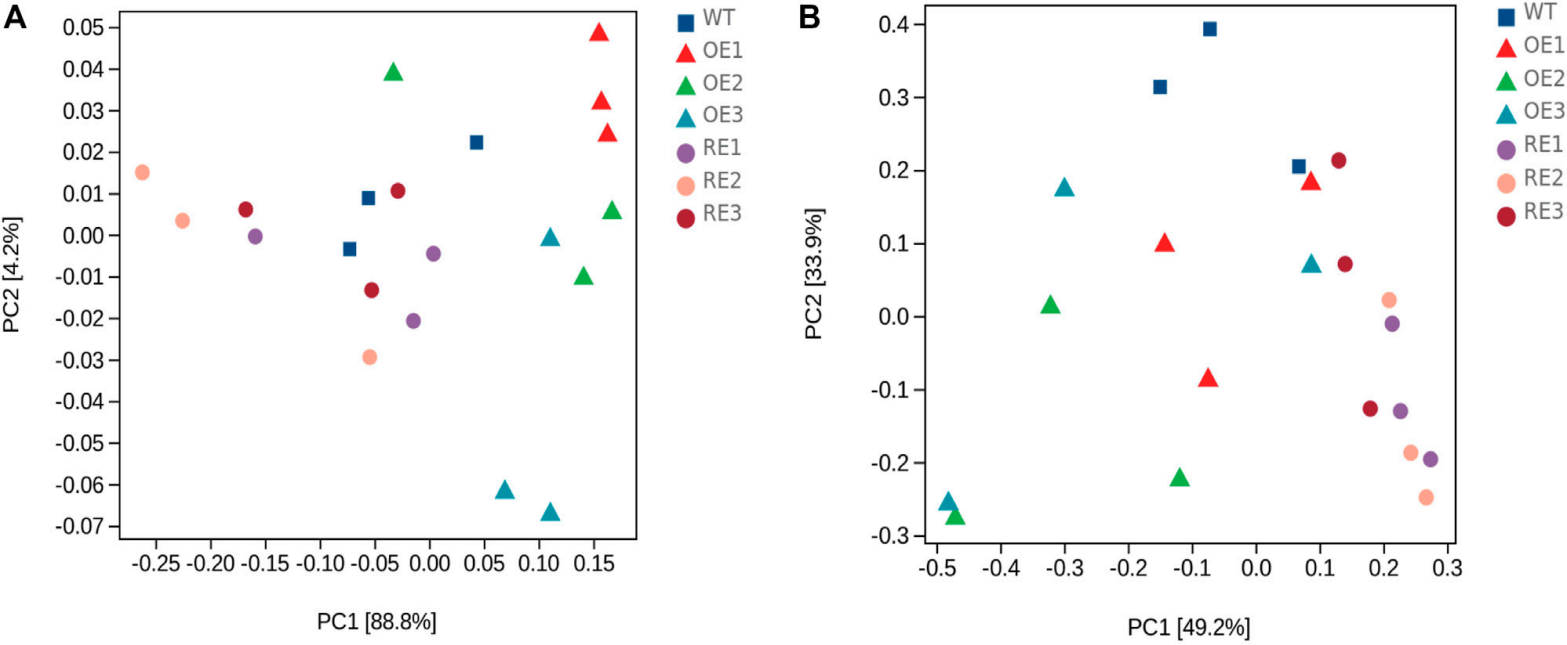
PCA of bacterial and fungal community genera in rhizosphere soil between transgenic *PaGLK* and WT poplar. **(A)** Bacteria **(B)** Fungi.

Principal component analysis (PCA) was performed at the ASV level, and the results showed that PC1 and PC2 to the bacterial community structure in rhizosphere soil were 88.8% and 4.2%, respectively (Figure 4A), and the cumulative contribution rate was 93.0%, which was considered to be the main reason for the change of bacterial community structure. As for fungal community in rhizosphere soil, PC1 and PC2 were 49.2% and 33.9%, respectively (Figure 4B), and the cumulative contribution rate was 83.1%, indicating that the above principal components could also explain the differences in fungal community. Figure 4 showed that rhizosphere soil bacterial composition difference between RE lines and WT lines was small. Fungal community composition difference between OE lines and WT lines was small as well.

**FIGURE 5:**
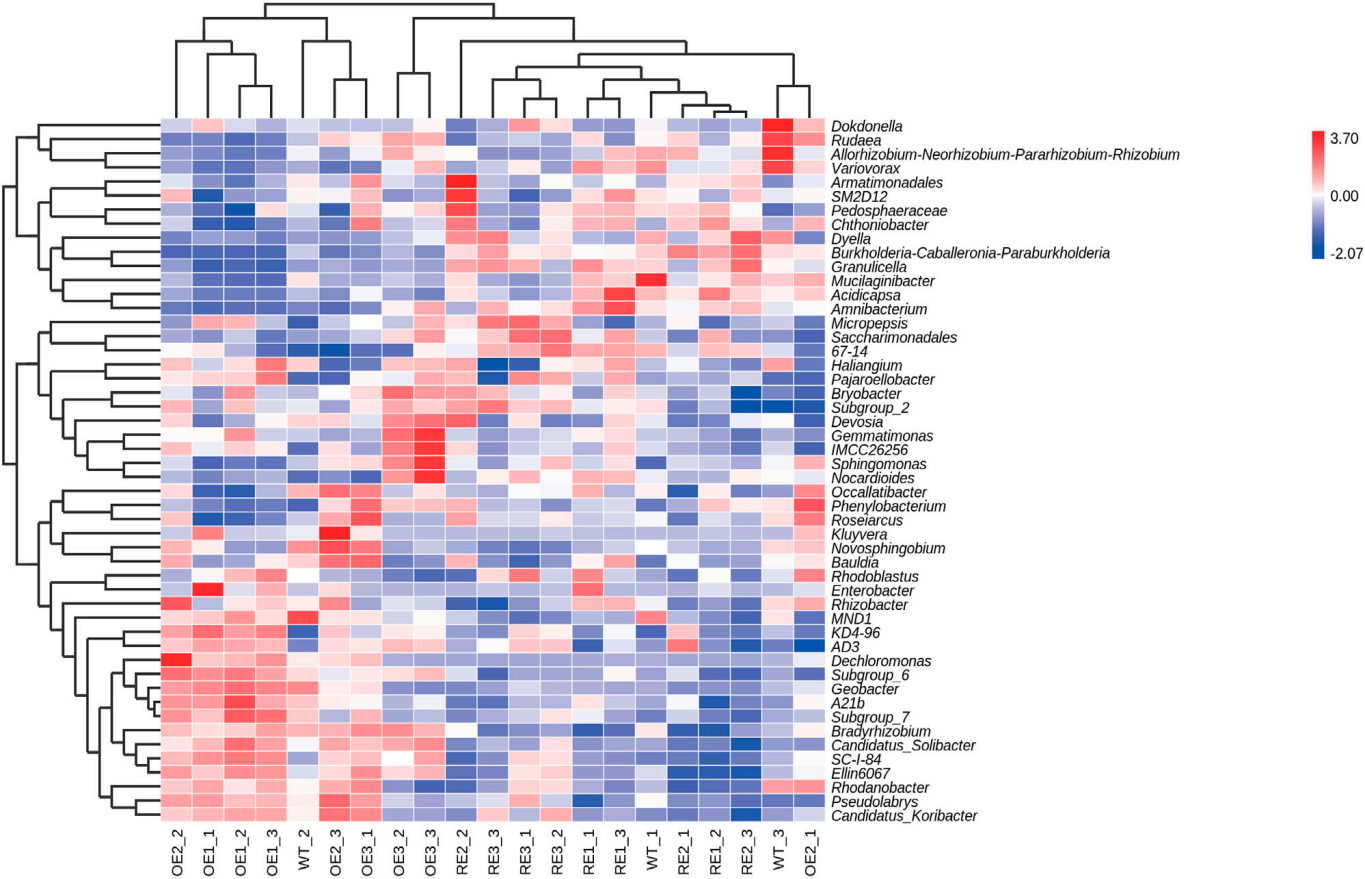
Abundance clustering heat map of the top 50 bacterial community genera in rhizosphere soil of the test lines.

**FIGURE 6:**
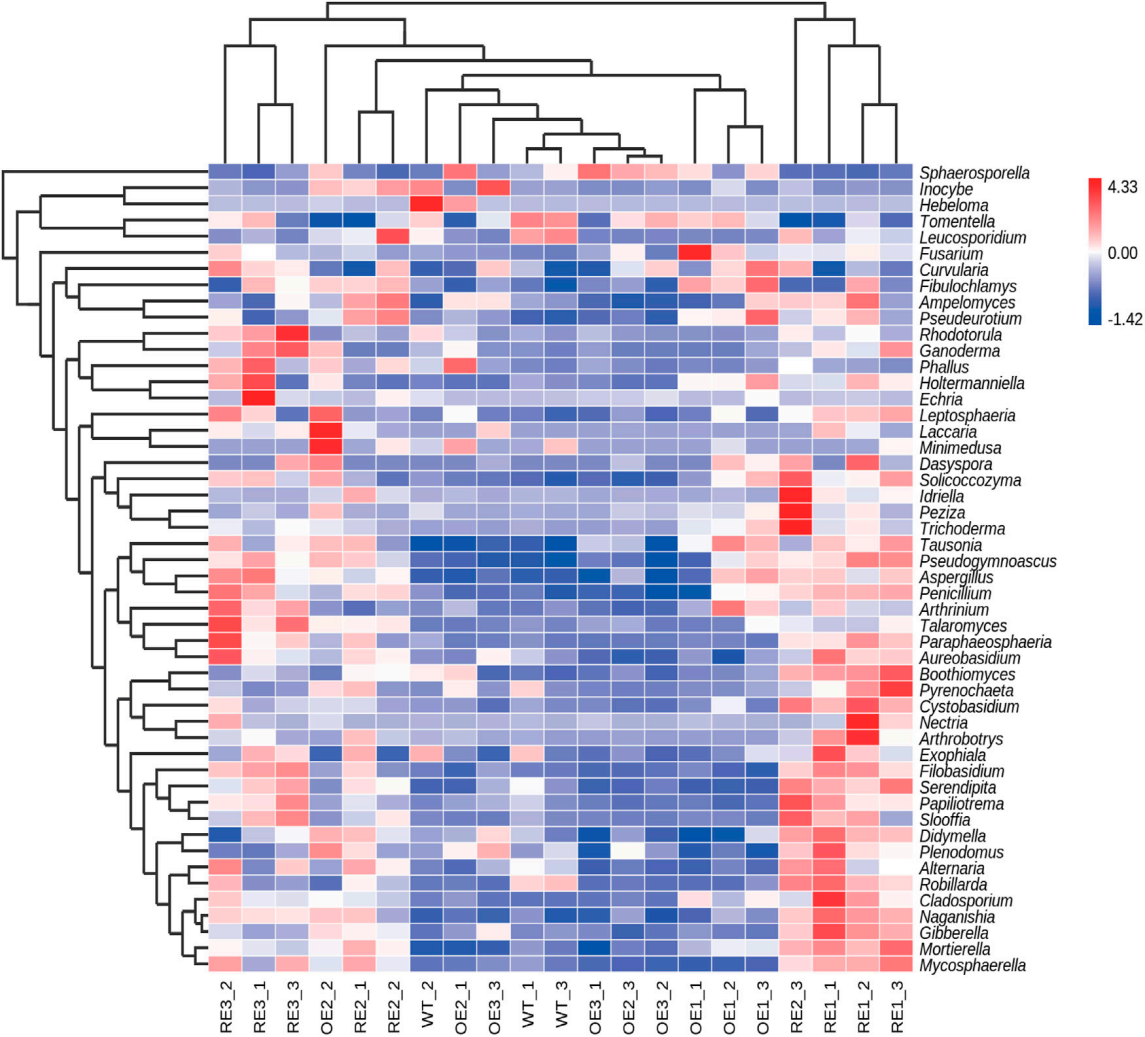
Abundance clustering heat map of the top 50 genera in rhizosphere soil fungal communities of the tested lines.

In order to further compare the horizontal composition of bacteria and fungi in rhizosphere soil between samples, the heat map was drawn by the average abundance of the top 50 genera. The results showed that RE lines was closer to WT lines in the rhizosphere soil bacterial (Figure 5) and OE lines were closer to WT lines in the rhizosphere soil fungi (Figure 6). The heat map results were consistent with the above PCA analysis.

### Different analysis of root exudates in transgenic *P.alba × P.berolinensis*

**FIGURE 7:**
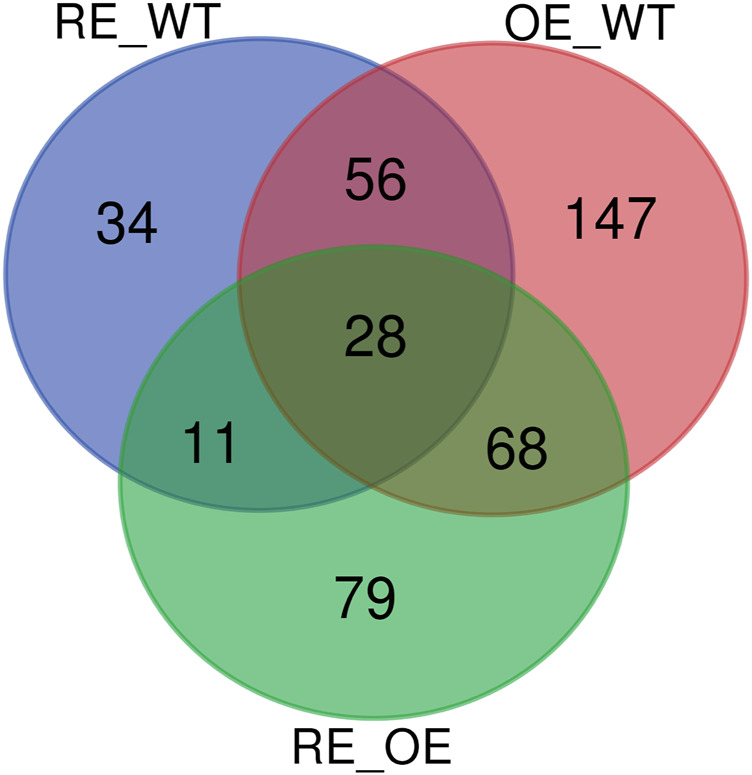
Wayne diagram of differential metabolites in three groups.

Figure 7 showed that the Wayne diagram based on the differential metabolites of the three groups. There were 28 common differential metabolites in the comparison 3 groups of RE vs. WT, OE vs. WT and RE vs. OE. Among them, the contents of 5 differential metabolites were significantly up-regulated in RE lines, but significantly down-regulated in OE lines. In addition, Among the other 23 different metabolites, 2 were significantly up‐regulated and 21 were significantly down regulated in transgenic lines (*p* < 0.05).

## Discussion

### Effects of transgenic poplar on soil enzyme activity

Soil enzymes are mainly produced by soil microorganisms and plant roots. Their activity reflects the dynamic change of organic matter in soil and the material cycle of C, N, S, P and other elements, which is an important indicator for evaluating soil quality (Sobucki et al., 2021). Rhizosphere is a key area for the interaction among plants, soil and microorganisms. Exogenous gene products of transgenic plants may enter the soil through root exudates or plant residues (Melnitchouck et al., 2006). Therefore, people tried to understand the impact of exogenous gene expression products on soil microecology through the changes in rhizosphere soil enzyme activities of transgenic plants. Studies showed that transgenic *Bt* cotton had no significant effect on soil urease (Shen et al., 2006; Luo et al., 2017), while some *Bt* lines had certain effect on soil urease. The degree of influence varied from cotton varieties, growth stages and soil types (Li et al., 2019). The rhizosphere soil enzyme activity of transgenic *PaGLK* poplar were different between transgenic lines and WT lines in different growing stages. As plant was growing, the rhizosphere soil urease, catalase and neutral protease activities of the tested lines showed an increasing trend (Figures 1B–D). However, cellulase showed a downward trend (Figure 1E). The rhizosphere enzyme activities of transgenic lines and WT lines at different growing stages were not the same (Figures 1B–D). For example, in mid-September, the activities of catalase and cellulase in rhizosphere of RE lines were higher than those of WT lines (Figure 1C); the activity of neutral protease in rhizosphere of OE lines was higher than that of WT. The other four enzyme activities were lower or significantly lower than that of WT lines. In this experiment, the rhizosphere soil enzyme activity of 2‐year‐old old transgenic lines was studied. Further long term study is needed to investigate whether the introduction of target genes and exogenous genes had an effect on rhizosphere soil enzyme activity.

### Effects of transgenic plants on soil microbial community

Studies have shown that the composition and richness of rhizosphere soil microbial community were often affected by plant species, plant growth period, tillage management and exogenous gene introduction (Gabriele Berg, 2009). For example, the study of herbicide-resistant transgenic soybean NZL06-698 on soil microbial community composition showed that its microbial species abundance and diversity decreased, as well as the abundance of nitrogen-fixing bacteria (Lu et al., 2017); Analysis of bacterial Alpha diversity and community structure in rhizosphere soil of transgenic *Bt* maize and non-transgenic maize at mature stage showed that there were significant differences in alpha diversity and community composition between them (van Wyk et al., 2017). Studies have shown that the composition and richness of soil microbial community in plant rhizosphere are often affected by biological and abiotic factors such as plant species, plant growth period, cultivation management mode and introduction of foreign genes. In this study, results showed that the fungal community abundance and diversity of OE and RE lines were higher than those of WT lines. There was no significant difference in bacterial community abundance and diversity between OE and WT lines, while RE lines was lower or significantly lower than WT.

Studies have also shown that although transgenic plants have a certain impact on rhizosphere soil microbial communities, but the impact may be less than the plant growth, fertilizer and field management and other factors (Liu et al., 2005; Susanne and Christoph C, 2005). For example, the rhizosphere microbial community of transgenic glufosinate-tolerant oilseed rape ( *Brassica napus*) did not change significantly compared with wild type, and the effect of different growing stages of rape on rhizosphere soil microbial community structure was greater than effect of transgenic plants (Gyamfi et al., 2002). Similarly, the results of transgenic *Bt* maize indicate that spatial variation and heterogeneity in the field had a greater effect on the community structure of the Arbuscular mycorrhizal fungi (AMF) than host *Bt* transgenic plant cultivars (Cheeke et al., 2015). In this study, we analyzed the rhizosphere soil bacterial and fungal communities of 2-year‐old transgenic *P. alba × P. berlinensis*. For perennial trees, whether these differences changed or weakened in later years needs further study. Our results showed that *Burkholderia* was the dominant bacterial genus in the rhizosphere of *P. alba × P. berlinensis*, and its relative abundance decreased in *PaGLK* OE lines (containing hygromycin resistance gene), and increased in *PaGLK* repression of RE lines (containing herbicide resistance gene). *Burkholderia* is a kind of bacteria with wide distribution and diverse functions. In recent years, with the increase of studies on plant-related *Burkholderia*, more and more evidence has shown that most species in the genus *Burkholderia* are important plant rhizobacteria. For example, involved in bioremediation, nitrogen fixation, growth promotion, induction of plant resistance, etc (Depoorter et al., 2016). ITS sequencing analysis showed that *Tomentella* was the dominant group in rhizosphere soil fungal community. The previous study has proved that the fungi of Tomentella are widely distributed in the world, which can form exogenous mycorrhizal fungi with *Salicaceae*, *Betulaceae*, and *Pinaceae* (Jakucs et al., 2005). The results of this study show that *Tomentella* is also a dominant fungal group in the rhizosphere of *P. alba × P. berlinensis*, and the relative abundance is the highest in the rhizosphere of WT lines.

### Effects of soil microorganisms on plant growth

Studies have found that rhizosphere microorganisms also change the physiology of plants or promote their growth in different ways. For example, *Serendipita indica* is a root fungus colonized in a variety of plants. It not only establish symbiotic relationships with plants, but also induce plants to synthesize secondary metabolites, thereby promoting plant growth, accelerating plant uptake of nutrients such as nitrogen and phosphorus, enhancing plant tolerance to adversity, and inducing plants to produce systemic resistance (Ray et al., 2021; Ray et al., 2021). This study also had the colonization of *Serendipita*, but the relative abundance of *Serendipita* fungi in the rhizosphere of RE lines was significantly higher than that of WT and OE lines (Figure 2B ). The RE lines’ leaf chlorophyll content is significantly lower than that of WT and OE lines. The 2-year-old plant height and ground diameter survey showed that the three RE lines did not affect plant height growth due to the decrease of leaf chlorophyll content. Whether the growth performance of RE lines was promoted by the distribution of highly abundant *Serendipita* fungi in the rhizosphere needs further verification.

### Effects of root exudates of transgenic poplar on microbial composition

The content of metabolites secreted by plant roots is closely related to ecological factors such as environment and climate conditions, and genetic factors also have a great impact on it (Calvo et al., 2019). In order to overcome the influence of environmental factors, root exudates were collected in sterile test tubes with the same culture conditions. OE lines can constitutively express *PaGLK* gene and hygromycin resistance gene (the protein kinase expressed by this gene can make hygromycin inactive), while RE lines decreased the *PaGLK* expression and the *BAR* gene product was expressed. Due to the difference of *PaGLK* gene expression level and the difference of marker genes, LC-MS analysis showed that the contents of (S) -ACPA, geniposidic acid, agnuside, hydroquinone and pyranocoumarins in RE lines were significantly higher than those in WT lines, while in OE lines, the contents of these substances were significantly lower than those in WT lines (Table 2). In the research system of the interaction between plants and rhizosphere microorganisms, the microorganisms that are attracted by plant root exudates and gathered around the rhizosphere are beneficial to plant growth and development , which can help plants obtain nutrients in extreme environments to help plants grow better (Mukhtar et al., 2019), there are also plant root rot caused by *Fusarium oxysporum*, resulting in decreased plant nutrient uptake and death (Maryani et al., 2019). Pyranocoumarins, agnuside, hydroquinones, geniposidic acid and other metabolites in the root differential metabolites of *PaGLK* transgenic *populus* have a variety of physiological and pharmacological activities such as antioxidant, free radical scavenging and antibacterial (Patel and Patel, 2011; Rajarajeswari and Pari, 2011). Our results show that the increase of the above metabolites in the rhizosphere of RE lines may be beneficial to the proliferation of *Serendipita*, and the decrease of its content in the OE lines is a favorable environmental condition for the reproduction of *Sphaerosporella*.

**TABLE 2:** 5 differential metabolites between RE vs. WT and OE vs. WT.

Name	Class	RE_WT *p*-value	OE_WT *p*-value
(S)-ACPA	Amino acids, peptides	0.013	0.044
Geniposidic acid	Terpene glycosides	0.032	0.012
Agnuside	Terpene glycosides	0.040	0.025
Hydroquinone	Benzenediols	0.020	0.002
(9R,10R)-10-hydroxy-8,8-dimethyl-9-{[(2S,3R,4S,5S,6R)-3,4,5-trihydroxy-6-(hydroxymethyl)oxan-2-yl]oxy}-2H,8H,9H,10H-pyrano[2,3-h]chromen-2-one	Pyranocoumarins	0.047	0.020

Rhizosphere is the main place for plants to communicate with rhizosphere microorganisms. The results showed that the OE and RE lines of *P. alba × P. berlinensis* showed some differences in root exudates, rhizosphere soil enzyme activity, bacterial and fungal community composition due to the different expression levels of *PaGLK* gene and the difference in selection marker genes. Some metabolites with pharmacological activities such as antioxidant and antibacterial in root exudates were significantly increased in RE lines, while those in OE lines were lower than those in WT line, at the same time, it was found that the rhizosphere soil of RE lines was probiotic increased significantly. In conclusion, the transgenic lines involved in the plant growth stage did not adversely affect root exudates, rhizosphere soil enzyme activities and microbial community composition. Since we only studied one season, we can’t confidently determine whether this effect was due to the transgenic *P. alba × P. berlinensis* lines or plant growing stages, or temporary unknow factors. Therefore, it is necessary to study the rhizosphere soil microbial community structure and soil enzyme activity of the tested lines in different development stages and different years.

## Data Availability

The original contributions presented in the study are included in the article/Supplementary Material, further inquiries can be directed to the corresponding author.
